# Practicing Emotionally Biased Retrieval Affects Mood and Establishes Biased Recall a Week Later

**DOI:** 10.1007/s10608-016-9789-6

**Published:** 2016-06-08

**Authors:** Janna N. Vrijsen, Paula T. Hertel, Eni S. Becker

**Affiliations:** 1 Department of Psychiatry, Radboud University Medical Center, PO Box 9101, 6500 HB Nijmegen, The Netherlands; 2 Department of Psychology, Trinity University, San Antonio, TX USA; 3 Behavioural Science Institute, Radboud University Nijmegen, Nijmegen, The Netherlands

**Keywords:** Memory bias, Retrieval, Depression, Rumination, Cognitive Bias Modification

## Abstract

Cognitive Bias Modification (CBM) can yield clinically relevant results. Only few studies have directly manipulated *memory* bias, which is prominent in depression. In a new approach to CBM, we sought to simulate or oppose ruminative processes by training the retrieval of negative or positive words. Participants studied positive and negative word pairs (Swahili cues with Dutch translations). In the positive and negative conditions, each of the three study trials was followed by a cued-recall test of training-congruent translations; a no-practice condition merely studied the pairs. Recall of the translations was tested after the training and after 1 week. Both recall tests revealed evidence of training-congruent bias and bias was associated with emotional autobiographical memory. Positive retrieval practice yielded stable positive mood, in contrast to the other conditions. The results indicate that memory bias can be established through retrieval practice and that the bias transfers to mood and autobiographical memory.

## Introduction

Depression is characterized by sustained negative affect and diminished positive affect. Emotional regulation problems are at the core of these depressive symptoms. Biased processing of emotional information affects emotional regulation and in turn maintained high negative affect and low positive affect (for reviews see Gotlib and Joormann 2010; Joormann and Quinn 2014; Mathews and MacLeod 2005). Under conditions in which never-depressed individuals give priority to positive information, depressed individuals tend to preferentially process negative information (Gotlib and Joormann 2010). Strong evidence exists that depression is marked by *memory* bias (Gaddy and Ingram 2014; Gotlib and Joormann 2010; Mathews and MacLeod 2005; Matt et al. 1992; Ridout et al. 2009). Remembering more negative (and/or fewer positive) events contributes to the onset and maintenance of depressive symptoms (e.g., Johnson et al. 2007; Rinck and Becker 2005). The experimental manipulation of cognitive biases (typically called Cognitive Bias Modification or CBM) attempts experimental control over the biases that are merely assessed in studies that measure performance by different groups or at different points in time. Such cross-sectional and longitudinal studies do not inform us about causality, which CBM does.

Studies on CBM use repeated practice in cognitive tasks and have succeeded in changing biases in attention and interpretation (Beard et al. 2012; Hertel and Mathews 2011; Menne-Lothmann et al. 2014). A few CBM experiments have modified interpretation biases in ways that affect subsequent measures of memory (e.g., Hertel et al. 2014; Joormann et al. 2015; Tran et al. 2011). However, more direct attempts to modify memory are rare (see Fox et al. 2014). Given the prominent role of memory bias in depression CBM-Memory seems a promising direction for CBM research.

Recently, Vrijsen et al. (2014) attempted to modify learning strategies by interjecting a series of study/test trials between pre-and post-tests of free recall of positive and negative words. On each trial, participants studied a new set of 10 positive and 10 negative words and then took a test of recall cued by the fragments of either the positive or the negative words (consistently across all training sets). Although no evidence of training-congruent free recall was obtained, in one study negative training produced proportionally more incorrect negative words or false memories. Thus, the negative CBM-Memory training either increased accessibility of training-congruent emotional information or, more generally, sensitivity to emotional aspects of experience.

The experiments by Vrijsen et al. (2014) were designed to test the prediction that the effect of the fragment tests during training trials would carry over to the free recall post-test. In contrast, the current experiment was focused more directly on retrieval practice. More specifically, retrieval practice may serve as a model for memorial aspects of depressive rumination and is therefore a promising target for CBM. Bringing negative thoughts to mind repeatedly is a feature of ruminative thinking. In rumination, essentially nonemotional cues can repeatedly prompt the retrieval of negative experiences that, in turn, invite perseverative, introspective focus on one’s negative thoughts and feelings (Nolen-Hoeksema et al. 2008). In short, repetitive retrieval of negative information is key to depressive rumination and hence a promising process to use in CBM.

Retrieval practice is not only a process of interest because of its association with rumination; basic cognitive psychological research consistently showed that retrieval practice also serves to enhance performance on later tests of memory. Karpicke and Roediger (2008) have shown that repeated tests but not repeated study episodes facilitate recall a week later. Each retrieval opportunity influences the later recall of the material (see for review Roediger and Butler 2011). Furthermore, retrieval practice and not study produces deeper learning of the material so that the learned information can be transferred to new contexts (Roediger and Butler 2011). This line of research has used texts and other material, such as Swahili–English word pairs, to show the advantage of retrieval practice for later recall. Retrieval practice might thereby help explain the development of negative memory bias in depression: Repetitively retrieving negative material from memory during rumination should facilitate superior recall of negatively (and not positively) biased memories (see Hertel et al. 2016). Each episode of retrieval increases the likelihood of future retrieval and likely contributes to the habit of rumination. In addition, the very act of retrieval provides additional exposure to the memory and encourages further attention and elaboration during future encounters or presentations (see the review by Karpicke and Grimaldi 2012).

In the current experiment, we examined the effect of retrieval-based memory training on immediate and delayed recall of Dutch translations of positive and negative words presented in Swahili. The retrieval-training design employed by Karpicke and Roediger (2008) was adapted to examine valence-specific retrieval training and transfer effects. Training was used to simulate biases in retrieval practice that seem to characterize either a ruminative (in the negative training condition) or a resilient thinking style (in the positive training condition). Participants studied Dutch translations of negative and positive Swahili words. In one experimental condition, translations of only the negative Swahili words were tested, and in another condition only the translations of the positive Swahili words were tested. The performance in these two conditions was compared to performance in a no-practice control condition in which participants merely studied the word pairs three times. This condition allowed for the examination of nonmanipulated memory bias. Recall of half of the negative and positive translations, cued in Swahili, was tested immediately after the three training trials, and recall of all translations was cued a week later. We expected training-congruent biased recall on the immediate test and predicted that the retrieval-practice effect would extend to the delayed test. In addition to predicting that the retrieval-practice affects memory bias, we investigated the effects of retrieval training on mood. We also explored the possibility that training would transfer to other forms of memory bias, including the emotionality of a recalled life event as a measure of real-world memory bias and the emotionality of intrusions (false recall) as a measure of a general bias in responding with a negative set. The training effects were predicted to transfer to mood, intrusions, and bias in autobiographical recall—an ecologically valid assessment of memory bias.

## Method

### Participants

A total of 95 undergraduate students from Radboud University Nijmegen participated in this study. Data from two participants were unavailable due to technical difficulties, resulting in a final sample of 93 participants. Students who had a Beck Depression Inventory (BDI-II; Beck et al. 1996; Dutch translation, van der Does 2002) total score higher than 28 were not invited for the study because we did not want to expose participants with severe depressive symptom levels (Beck et al. 1996) to the negative training condition. Participants were randomly assigned to one of the three conditions of retrieval practice: Negative (*n* = 31), no-practice (*n* = 31), or positive (*n* = 31). Descriptive statistics are presented in Table 1. The three groups did not differ in age, sex, medication use (0 % negative, 7 % no-practice, 3 % positive, χ^2^(93) = 2.07, *p* = .36), or past psychological treatment (19 % in all groups). Neither did the groups differ according to their scores on the BDI-II, the Beck Anxiety Inventory (BAI-II; Beck et al. 1988), or the Ruminative Response Scale (RRS; Nolen-Hoeksema and Morrow 1991; Dutch translation, Raes et al. 2003). All participants were native Dutch speakers and received course credit for their participation. At sign-up, and both at the start and end of the lab session, participants were informed that the study had two phases: a lab session and an online 1-week follow-up session.

**Table 1:** Percentages or means (SD) on demographic and assessment measures

	Training condition			
	Negative	No-practice	Positive	*F*(2, 90)
Age (years)	19.32 (0.35)	19.84 (0.35)	19.84 (0.35)	0.73, *p* = .48, *f* = .13
Sex (%female)	77 %	84 %	77 %	χ^2^(2, N = 93) = 0.53, *p* = .77
BDI-II	5.06 (4.32)	7.74 (6.95)	5.29 (4.85)	2.27, *p* = .11, *f* = .22
BAI-II	31.00 (6.14)	31.00 (5.86)	29.32 (7.85)	0.65, *p* = .52, *f* = .12
RRS	37.68 (7.96)	41.45 (13.03)	36.77 (9.54)	1.77, *p* = .18, *f* = .20
RRS brooding	8.26 (2.19)	9.32 (3.58)	8.97 (2.42)	1.17, *p* = .32, *f* = .16
RRS reflection	8.87 (3.14)	8.58 (2.95)	7.87 (3.32)	0.83, *p* = .44, *f* = .14

BDI-II refers to the score on the Beck Depression Inventory, BAI-II to the Beck Anxiety inventory, RRS to the total score on the Ruminative Response Scale, RRS brooding to the 5-item brooding subscale, and RRS reflection to the 5-item reflection subscale (Treynor et al. 2003)

### Materials

#### Questionnaires

The BDI-II is a depression severity questionnaire consisting of 21 items, each rated 0–3 according to severity of difficulties experienced. Scores are summed; depression can then be interpreted as minimal (0–13), mild (14–19), moderate (20–28), or severe (≥ 29; Beck et al. 1996).

The RRS includes 22 items each rated 1–4 describing responses to depressed mood. Responses can be acting or thinking about the depressive symptoms and thinking about possible causes and consequences of the sad/depressed mood. A total score can be calculated, summing all 22 items scores. To examine correlations of emotional recall measures with rumination, we scored the RRS subscales of brooding and reflective pondering (Treynor et al. 2003), which are both related to depression and biased processing (Joormann et al. 2006). Brooding is considered to be the more pathological form of rumination, whereas reflective pondering is seen as “a purposeful turning inward to engage in cognitive problem solving to alleviate one’s depressive symptoms” (Treynor et al. 2003, p. 256).

The BAI consists of 21 items scored 0–3 and meaures overall anxiety levels. Scores are summed. This questionnaire was included to rule out possible difference on anxiety between the training condition groups. The BDI-II, RRS, and BAI questionnaires have good psychometric properties (respectively: van der Does 2002 (Dutch translation); Raes et al. 2003 (Dutch translation); Beck et al. 1988).

#### Stimuli

Forty Swahili words (20 with emotionally negative and 20 with positive meanings) were balanced across valence and subsets intended for immediate testing on length of the Dutch translations and frequency in the Dutch language (from the Dutch translation of Affective Norms for English Words (ANEW); Bradley and Lang 1999). Words were selected on valence strength to yield pronounced differential training effects. This set included depression-specific and general negative and positive words (e.g., *cruel, guilt, suicide, cheerful, friendschip, laughter*). Perceived emotional valence scores for the Dutch translations (Arnold et al. 2011) ranged from 1 (very negative) to 15 (very positive); (*M*_negative_ = 2.9, *SD* = .64; *M*_positive_ = 13.0, *SD* = .40).

#### Mood Ratings

**Fig. 1:**
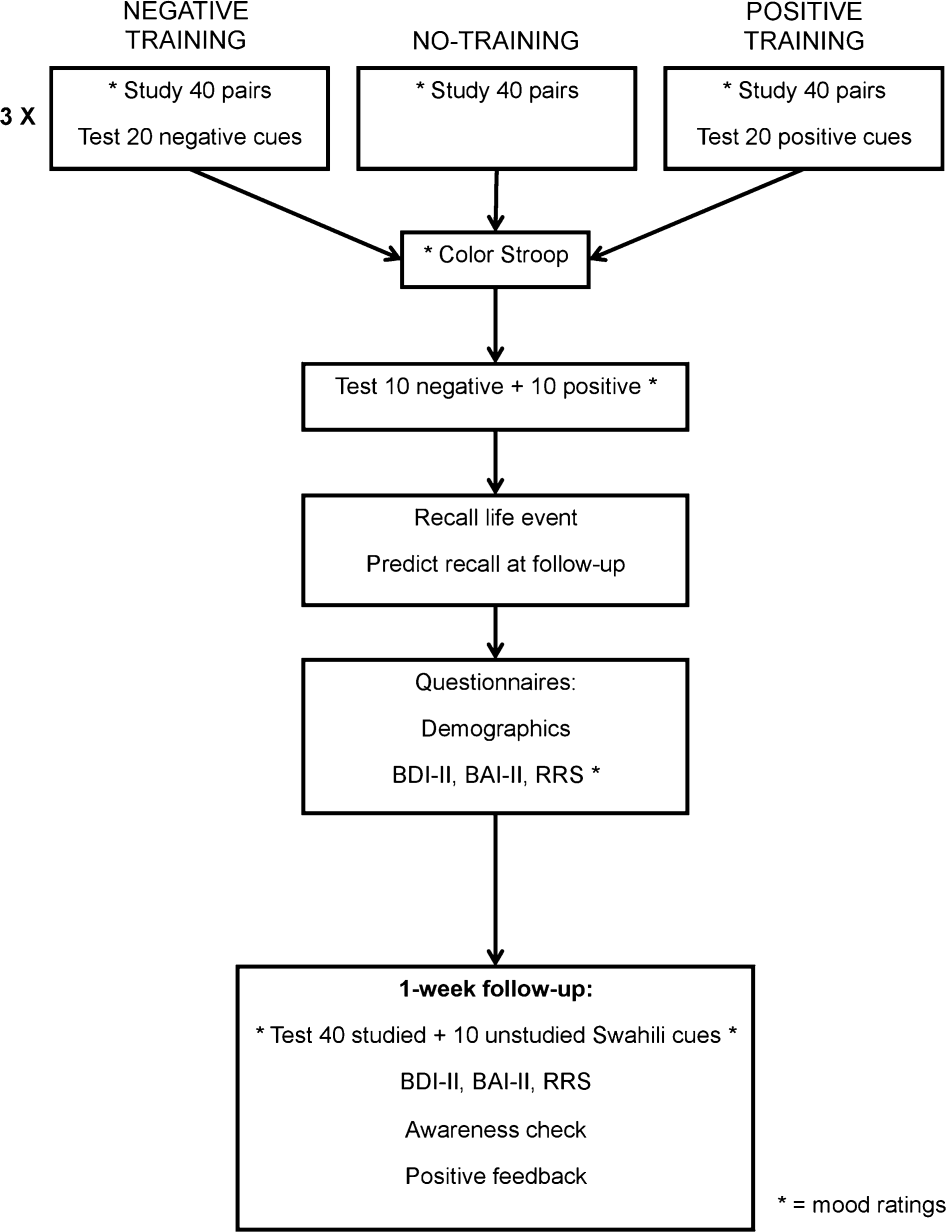
Schematic overview of the experiment. Pairs consisted of Swahili cues and Dutch translations as targets; half were emotionally negative and half positive words

Throughout the sessions, we assessed positive mood (*How positive, happy, or good do you feel right now?*), negative mood (*How negative, sad, or bad do you feel right now?*), relaxation, anxiety, and avoidance, in the order just reported. Such scales are a reliable, simple, and rapid was to assess mood and anxiety state (Abend et al. 2014; Cella and Perry 1986; Rossi and Pourtois 2013). Each question was followed by a 9-point Likert scale ranging from ‘not at all’ to ‘extremely’. The first two scales were the ones of interest; others were included to make that fact less obvious. Figure 1 shows when mood was assessed.

### Procedure

Participants were recruited for two sessions, scheduled 1 week apart. Session 1 assessed mood at the start, following training, following the end-of-session test of 20 training-congruent translations, and following the questionnaires. Session 2 was conducted online and began and ended with a mood assessment; the follow-up test presented all 50 Swahili words, including the 10 nonstudied (new) words intended to test a general recall bias. Figure 1 depicts the exact order of the procedure. Informed consent was obtained for all participants.

#### Session 1 Training

Retrieval training consisted of three study/test trials. During the study portion of each trial, all participants viewed the 40 Swahili-Dutch word pairs (20 with a positive and 20 with a negative translation), each presented in white in the middle of a black computer screen for 10 s, with a 500-ms intertrial interval. The order of the words was randomized, with the constraint that no more than two words of the same valence were presented consecutively, and was kept constant for all participants. Participants were instructed to memorize the word pairs. After a 30-s distraction (by simple arithmetic calculations), participants in the negative and positive training conditions were presented with the 20 training-congruent Swahili words, each in turn, in the same order used in the study phase. Participants attempted to type the Dutch translation during the 8 s allotted for responding. The typed response appeared in yellow below the Swahili word. No direct performance feedback was given (i.e. errors/correct responses were not indicated), but the subsequent study trial provided indirect feedback for the first and second of the practice tests. The duration of a study trial was approximately 7 min and a test trial approximately 3 min. Test trials were withheld from participants in the no-practice condition; they merely studied the 40 word pairs and performed the distraction task in between the three study trials. To eliminate any possible mood effect induced by retrieval practice and to control for possible recency effects, the final study/test trial of the training phase was followed by a brief version of the Stroop task that lasted approximately 2 min.

#### Session 1 Assessment: Immediate Test, Autobiographical Recall, Prediction of Recall, and Questionnaires

After the Stroop distraction, we tested recall of half of the translations. All participants saw 10 positive and 10 negative Swahili words for 10 s each and were instructed to type each translation within 10 s. Two fixed subsets of cues were created by randomly assigning words from the original list, and the role of the subsets (tested in Session 1 or not) was counterbalanced within each training condition. Following this test, autobiographical recall was assessed by asking participants to recall a personal life event that made an impression on them and to type its description. Instructions were deliberately simple and broad to potentiate transfer of training (see Hertel and Mathews 2011). Next, following the procedure established by Karpicke and Roediger (2008), they predicted how many of the 40 word pairs they would correctly recall after 1 week. At the end of Session 1 they answered demographic questions and responded to the BDI-II, BAI-II, and RRS, in that order.

#### Session 2

Exactly 1 week after Session 1, participants received an email with a link to an online follow-up task and a personal code to log in. Because we wanted to assess training effects in a natural surrounding we used an online program for Session 2. This meant participants did not have to come into the lab and could do this assessment from home. Following the mood assessment, they were presented with 50 Swahili words, including the 40 that they studied a week earlier and 10 unstudied words to assess the emotional bias in confabulations.^1^ They were instructed to type each translation within 15 s (in line with Karpicke 2009). The order of the words was the same as during Session 1, with the 10 unstudied words intermixed and the constraint of no more that two consecutive words in each valence; no time limit was set for responding. Following a final mood assessment, participants filled out the BDI-II, the RRS, and an awareness check.^2^ Positive performance feedback was provided to counteract possible negative mood effects from the training. Participants were then debriefed and received course credit in return for their participation.

## Results

In scoring correct recall, spelling errors as well as plurals if the words were singular (and vice versa) were permitted. When significant lower-order effects were qualified by significant higher-order interactions, they are not reported. The significance level was set conventionally at .05.

### Session-1 Recall

The percentage recalled (out of 10) on the end-of-session test was submitted to an analysis of variance (ANOVA) with a between-subjects factor for training condition (negative, no-practice, positive) and a within-subjects factor for valence (negative, positive). Cohen’s standards for the interpretation of partial eta squared effect size were used (1988): Small (.01), medium (.06), and large (.14). The interaction was significant, *F*(2, 90) = 11.89, *MSE* = 148.50, *p* < .001, η_p_^2^ = .21. Power calculation using G*power (Faul et al. 2007) revealed a power of .99 for this interaction effect. The training conditions differed in recalling negative words, *F*(2, 90) = 4.83, *MSE* = 557.63, *p* = .010, η_p_^2^ = .10. This simple main effect was further understood by conducting a *t* test for each pair-wise comparison. Negative practice produced higher levels of negative translations on this test, compared to the no-practice and positive conditions (*M* = 71.9, *SD* = 24.1 for the negative training condition, *M* = 55.4, *SD* = 24.1 for no-practice, and *M* = 56.1, *SD* = 22.6 for positive), with *t*(60) = 2.69, *SE* = 6.12, *p* = .009 comparing negative to no-training, and *t*(60) = 2.66, *SE* = 5.94, *p* = .010 for the negative compared to the positive training condition. There was no significant difference in recall between the no-practice and positive conditions, *t*(60) = 0.11, *SE* = 5.93, *p* = .911.

The training conditions also differed significantly in recalling positive words, *F*(2, 90) = 3.68, *MSE* = 608.32, *p* = .029, η_p_^2^ = .08. Positive practice produced higher levels of translated positive words compared to the no-practice condition (*M* = 69.7, *SD* = 23.7, vs. *M* = 53.2, *SD* = 26.6), *t*(60) = 2.57, *SE* = 6.41, *p* = .013. The negative training condition (*M* = 65.2, *SD* = 23.5) resulted in marginally higher levels of positive recall than the no-practice condition, *t*(60) = 1.87, *SE* = 6.38, *p* = .066. However, the percentage of positive recall did not differ significantly between the positive and negative training conditions, *t*(60) = 0.75, *SE* = 5.99, *p* = .455.

### Session-2 Recall

**Fig. 2:**
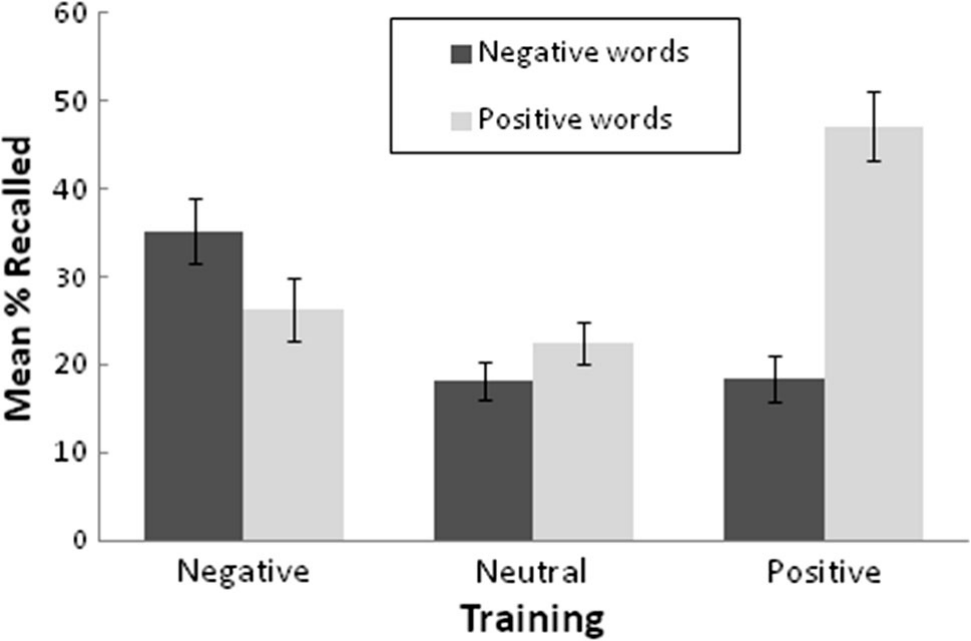
Mean percent of translations recalled on the delayed test. *Error bars* 1 SE

The percentage recalled (out of 10) on the follow-up test was submitted to an ANOVA with a between-subjects factor for training condition (negative, no-practice, positive) and within-subjects factors for valence (negative, positive) and whether the cue had been tested at the end of Session 1 (tested, untested). There was a main effect of testing, as tested cues produced overall higher levels of recall in Session 2 (*M* = 33.7, *SE* = 1.9, vs. 22.2, *SE* = 1.6 for untested cues), *F*(1, 90) = 97.93, *MSE* = 124.53, *p* < .001, η_p_^2^ = .52. A nonsignificant trend for the three-way interaction was obtained, *F*(2, 90) = 2.49, *MSE* = 136.51, *p* = .089, η_p_^2^ = .05, however the interaction between training condition and testing status was nonsignificant when examined for negative and positive words separately, *p* > .10 in both cases. Regardless of prior testing, the interaction between training condition and valence was significant, *F*(2, 90) = 54.34, *MSE* = 207.65, *p* < .001, η_p_^2^ = .55; see Fig. 2. The simple main effect of training condition was significant for recall of positive as well as negative words; *p* < .001 in both cases. As with the Session-1 results, this effect was explained by conducting a *t* test for each pair-wise comparison. Negative practice produced higher levels of translated negative words, compared to the no-practice and positive conditions, *t*(60) = 3.79, *SE* = 4.20, *p* < .001 and *t*(60) = 3.72, *SE* = 4.50, *p* < .001, respectively. The difference between the positive condition and the no-training condition was nonsignificant, *t*(60) = 0.24, *SE* = 3.35, *p* = .810. Positive practice produced higher levels of translated positive words, compared to the no-practice and negative conditions, *t*(60) = 5.06, *SE* = 4.59, *p* < .001 and *t*(60) = 3.90, *SE* = 54.25, *p* < .001, respectively. The negative condition and no-practice condition did not differ on level of translated positive words, *t*(60) = 0.65, *SE* = 4.21, *p* = .517. The interaction between training condition and valence explained an apparently higher proportion of variance in Session-2 recall than in Session-1 recall.

When comparing the Session 2 recall data to the data on initial recall, we see a main effect of Time showing lower levels of overall recall after 1 week, *F*(1, 90) = 254.99, *MSE* = 412.47, *p* < .001, η_p_^2^ = .74. The three-way ANOVA testing the interaction between training condition, valence, and time of testing (Session 1 or 2) was significant, *F*(2, 90) = 6.46, *MSE* = 86.00, *p* < .005, η_p_^2^ = .13. Regardless of training condition, successful recall decreased less for positive compared to negative words from Session 1 to Session 2, *F*(1, 90) = 11.92, *MSE* = 86.00, *p* < .005, η_p_^2^ = .12. Also, regardless of valence, forgetting was similar across conditions, *F*(2, 90) = 1.09, *MSE* = 412.47, *p* = .342, η_p_^2^ = .02.

A total of 85 participants responded to the question regarding their prediction for recall at Session 2; 8 participants did not provide an estimation of recall. The estimates did not differ significantly according to training conditions, *F*(2, 82) = 2.55, *MSE* = 34.61, *p* = .084, η_p_^2^ = .06. On average, participants in all conditions predicted they would recall about 25 % of the translations in Session 2, (*M* = 10.3 *SD* = 6.0). The correlation between predicted recall and actual correct recall after 1 week was significant, *r*(83) = .41, *p* < .001, in contrast to the lack of correlation obtained by Karpicke and Roediger (2008).

### Session-1 Autobiographical Recall

The descriptions of personal life events were scored by two independent raters, blind to training conditions, as being positive or negative, Kappa = .85, *p* < .001. Inconsistent scores were resolved by a third rater. Two participants did not provide a description. First the direct effect of the training on valence (positive or negative) of autobiographical recall was tested using a Chi-square test. This yielded nonsignificant results, χ^2^(2, N = 91) = 1.01, *p* = .581. Continuing our exploratory approach, we then examined whether the training condition, considered together with recall bias on the immediate test and the recent mood description, predicted the valence of autobiographical recall. Candidates for entering the logistic regression model for valence of autobiographical recall included the codes for training conditions, the positive mood rating collected prior to Session-1 recall, and the percentages of positive and negative translations recalled immediately prior to the autobiographical memory task. Using forced entry, only the two recall variables were significantly associated with the valence of the life-event description: The chance of recalling a positive event was larger if more positive translations were recalled and lower if more negative translations were recalled associated, Odds ratio 1.04, *p* = .029, and Odds ratio 0.96, *p* = .018, respectively. The full model explained 11.9 % of variance (Nagelkerke *R* ^2^) in the valence of autobiographical recall, but was not significant, χ^2^(5, N = 91) = 8.17, *p* = .147.

### Mood Changes

Session-1 ratings for positive and negative mood were separately submitted to ANOVAs, with a between-subjects factor for training condition and a within-subjects factor for time of administration (before training, after training, after the Session-1 test, end of session). No significant effects were obtained for negative ratings. For positive-mood ratings (see Table 2), the interaction was significant; *F*(6, 270) = 2.45, *MSE* = 0.885, *p* = .025, η_p_^2^ = .05. Ratings in the positive training condition remained stable and high throughout the session, *p* > .74), whereas those in the other two conditions declined. In the negative training condition, the nature of the decline showed a cubic trend, *F*(1, 30) = 19.02, *MSE* = 0.503, *p* < .001, η_p_^2^ = .39. Participants felt better after the opportunity to recall both positive and negative translations but worse again at the end of the session. In the no-practice condition (where recall was lowest on the Session-1 test), ratings fell after “training” and stayed at that level, as indicated by a significant quadratic function, *F*(1, 30) = 6.61, *MSE* = 0.738, *p* = .015, η_p_^2^ = .18.

**Table 2:** Means (SD) for positive mood ratings

	Training condition		
	Negative	No-practice	Positive
First	6.9 (1.5)	6.9 (1.2)	7.1 (1.4)
After training	6.2 (1.4)	6.2 (1.3)	6.9 (0.8)
After test	6.8 (1.1)	6.2 (1.6)	7.1 (0.7)
Last	6.0 (1.7)	6.4 (1.5)	7.2 (0.8)
FU: first	6.8 (1.3)	6.8 (1.4)	6.7 (1.4)
FU: after test	6.6 (1.4)	6.2 (1.4)	6.7 (1.3)

FU refers to the ratings on the 1-week follow-up session

Similar analyses were performed on Session-2 ratings. Again, no significant effects were obtained for negative ratings, but the interaction of time and training condition was significant for positive ratings (see Table 2), *F*(2, 90) = 3.70, *MSE* = 0.394, *p* = .029, η_p_^2^ = .08. Participants who had not practiced retrieval in Session 1 again showed a mood decline after the test, *F*(1, 30) = 7.84, *MSE* = 0.595, *p* = .009, η_p_^2^ = .21. Both positive and negative training conditions maintained their mood from the start of the session, all comparisons *p* > .21).

### Rumination-Related Recall

Correlations between measures of depressive symptoms/rumination and measures of recall were nonsignificant in the positive and negative training conditions (both *p* > .10). However, it was the condition of no retrieval practice that we had intended to assess. We reasoned that correlations in this condition might illustrate naturally occurring biases in recall. Within the condition of no retrieval practice, correlations between Session-1 positive recall and the measures of depressive symptoms and rumination were nonsignificant (*p* > .40). Instead the percentages of negative words recalled were positively correlated with the Session-1 RRS scores (*r* = .37, *p* = .041), as well as with the RRS reflection subscale (*r* = .43, *p* = .015).

In the no-practice condition in Session 2, none of the measures of depressive symptoms or rumination was significantly associated with negative-word recall (*p* > .29), but many were positively associated with positive-word recall. Among the stronger correlations were BDI-II in Session 1 (*r* = .45, *p* = .011), the Session-1 brooding subscale of the RRS (*r* = .42, *p* = .019), BDI-II in Session 2 (*r* = .51, *p* = .004), and the brooding score in Session 2 (*r* = .44, *p* = .014).

## Discussion

The effect of repetitive and biased retrieval on subsequent and delayed recall of both positive and negative material was examined using a novel emotionally biased retrieval training paradigm. Retrieval training was successful in producing biased recall: We found a moderate training-congruent effect on immediate recall and a strong effect on delayed recall. Biased retrieval of negative material seems to have produced a ruminative-like processing style, as evidenced by the negative memory bias on both immediate and delayed tests. In contrast, positive retrieval practice was only slightly evident on the immediate test, but it produced a larger effect on delayed recall. The positive processing bias generally found in healthy, well-functioning individuals as in our sample (see Gotlib and Joormann 2010; Matt et al. 1992) might not be manipulated as easily as negative bias in a one-session training. Although the association between retrieval training and rumination requires more attention, we believe that the data from this delayed test simulates the outcome of retrieval processing that is naturally associated with the tendency either to ruminate or to look on the bright side. Each episode of retrieval strengthens the probability that the target will come to mind in the future. It also provides the opportunity for additional operations that enhance learning (Karpicke and Grimaldi 2012; Soderstrom and Bjork 2015).

Performance in the no-practice condition allowed us to examine natural tendencies for learning to be associated with depressive state and ruminative style. We reasoned that correlations in this condition might illustrate naturally occurring biases in recall, knowing full well that such biases are often not obtained with dysphoric samples. We observed the expected association between rumination and immediate negative recall. An unexpected but interesting outcome, however, was that participants who experienced more depressive symptoms in Session 1 tended to recall more positive words on the final test. College students, such as our recruited participants, seem to have the ability or tendency to use positive recall to overcome dysphoric states and ruminative thinking (Joormann and Siemer 2004). This interpretation is consistent with the finding that positive retrieval practice maintained a positive mood, in contrast to the decrease in positive affect across tasks in the other conditions.

Mood remained stable and high in the positive training condition, whereas it declined in the other two conditions. Practicing the retrieval of positive translations seemed to protect participants from the sort of mood decline typically associated with imperfect performance. Recall was no more accurate in the positive training condition than in the negative condition, so it was the *nature* of retrieval practice and not recall success that preserved the positive mood state. Effects of training in cognitive tasks on subsequent measures of mood have been found with other CBM paradigms as well (Hertel and Mathews 2011). Similarly our mood results show that remembering positive translations preserves a positive mood. Such effects provide examples of far transfer of training, in that transitory changes in mood can result from attending, thinking, and remembering in biased ways. Also, and even though our results do not allow for substantiation, retrieval training might affect attentional processing during conscious retrieval (Everaert and Koster 2015).

Other evidence of transfer is found on tasks that use similar but not identical measures of the phenomenon undergoing modification. In our experiment, performance on the autobiographical task provided our measure of cognitive transfer. No direct effect of the training on the autobiographical memory task was found. However, biased recall in the experimental task (substantially incurred by the training manipulation) was significantly associated with the valence of the life event description in the autobiographical task. This provides indirect support for the transfer of the retrieval training. These results should be augmented by more findings of direct effects of training, and the examination of transfer of training effects across time should be pursued.

An unexpected outcome was that our data did not replicate the high level of performance often found in other demonstrations of retrieval-practice effects using Swahili vocabulary (e.g., Karpicke and Roediger 2008). Compared to these previous experiments, our words were likely more abstract and were not all nouns. Participants also were given less time to attempt recall of the translations in our experiment. In line with the lower levels of actual recall, however, our participants predicted lower levels of recall than did participants in the previous experiments. In addition, we found a positive correlation between predicted recall and actual correct recall after 1 week, whereas Karpicke and Roediger (2008) did not. Perhaps less than perfect initial performance provides a better basis for judging subsequent recall. Furthermore, our design does not permit an examination of whether practicing the retrieval of emotional translations would produce different outcomes from practicing the retrieval of neutrally valenced translations. In the current study, we assessed trait rumination. To measure the training effects on ruminative thinking, we intend to include a measure of state rumination in a future study. Important to note is that the current results provide first evidence in a healthy sample; clinical applicability and generalization of results to (sub)clinical samples requires further study. Another boundary condition for obtaining the current results relates to the repetition of study sessions as feedback for two of the practice tests. Corrective feedback enhances training effects (see Roediger and Butler 2011), but it strays from our goal of mimicking ruminative uses of memory. However, this feature of our design seemed necessary, given difficulties associated with learning Swahili. Future experiments with other materials should examine the importance of this feedback feature to the effects examined here.

## Conclusions

In conclusion, the strategy of practicing biased retrieval is effective in establishing recall biases a week later, perhaps even later than that. This study is part of a new direction in CBM research (Fox et al. 2014): The modification of biases in remembering, globally referred to as CBM-Memory. On the “negative” hand, retrieval practice might well be involved in ruminative episodes leading to depression; on the “positive” hand, it might be harnessed to oppose rumination. Both “hands” obviously require further support, beginning with evidence that naturally occurring recall biases in ruminators or depressed individuals can be reduced or eliminated through positive retrieval practice (see Hertel et al. 2016).

## Footnotes

1 An additional 10 Swahili words with Dutch translations were selected (5 with emotionally negative meanings and 5 with positive) with valence ratings comparable to the studied words (*M*_*negative*_ = 3.1, *SD* = 0.28; *M*_*positive*_ = 13.0, *SD* = 0.20). Only the main effect of valence was significant, *F*(1, 90) = 9.64, *MSE* = 72.26, *p* = .003, η_p_^2^ = .10; 10.2 % of the new cues produced positive responses (*SD* = 13.3) and 6.3 % produced negative responses (*SD* = 12.0). Retrieval practice did not reliably affect this generally positive bias, *p* = .13. Responses to the new cues were often correct translations of orthographically similar studied words. The positive bias might therefore represent only greater orthographic similarity between the new cues and the positive studied cues, compared to the similarity with negative studied cues.

2 At the end of the study, we asked three questions to assess participants’ awareness of the study aim: *What do you think this study was about? What do you think we aimed to measure? Did you notice anything in yourself during the study (a feeling, a learning strategy,* etc*.)?* Of the n = 57 who provided a serious answer to the awareness check (some just filled a question mark or random answer), 82.5 % of participants guessed that the study had something to do with mood and learning. Of those participants, 42.4 % thought it was about the association between learning positive and negative words and mood. Several participants thought it was about long-term versus short-term memory, no doubt due to the long interval between sessions. No one mentioned anything concerning a manipulation of learning strategies. Moreover, none of the participants reported that training influenced their autobiographical recall.

## References

[CR1] Abend R, Dan O, Maoz K, Raz S, Bar-Haim Y (2014). Reliability, validity and sensitivity of a computerized visual analog scale measuring state anxiety. Journal of Behavior Therapy and Experimental Psychiatry.

[CR2] Arnold JF, Fitzgerald DA, Fernandez G, Rijpkema M, Rinck M, Eling PA (2011). Rose or black-coloured glasses? Altered neural processing of positive events during memory formation is a trait marker of depression. Journal of Affective Disorders.

[CR3] Beard C, Sawyer AT, Hofmann SG (2012). Efficacy of attention bias modification using threat and appetitive stimuli: A meta-analytic review. Behavior Therapy.

[CR4] Beck AT, Epstein N, Brown G, Steer RA (1988). An inventory for measuring clinical anxiety: Psychometric properties. Journal of Consulting and Clinical Psychology.

[CR5] Beck AT, Steer RA, Brown GK (1996). Manual for Beck Depression Inventory-II.

[CR6] Bradley MM, Lang PJ (1999). Affective norms for English words (ANEW): Instruction manual and affective ratings.

[CR7] Cella DF, Perry SW (1986). Reliability and concurrent validity of three visual-analogue mood scales. Psychological Reports.

[CR8] Everaert J, Koster EHW (2015). Interactions among emotional attention, encoding, and retrieval of ambiguous information: An eye-tracking study. Emotion.

[CR9] Faul F, Erdfelder E, Lang AG, Buchner A (2007). G*Power 3: A flexible statistical power analysis program for the social, behavioral, and biomedical sciences. Behaviour Research Methods.

[CR10] Fox E, Mackintosh B, Holmes EA (2014). Travellers’ tales in cognitive bias modification research: A commentary on the special issue. Cognitive Therapy and Research.

[CR11] Gaddy MA, Ingram RE (2014). Meta-analytic review of mood-congruent implicit memory in depressed mood. Clinical Psychology Review.

[CR12] Gotlib IH, Joormann J (2010). Cognition and depression: Current status and future directions. Annual Review of Clinical Psychology.

[CR13] Hertel PT, Mathews A (2011). Cognitive bias modification: Past perspectives, current findings, and future applications. Perspectives on Psychological Science.

[CR14] Hertel, P., Maydon, A., Cottle, J., & Vrijsen, J. (2016). Cognitive Bias Modification: Retrieval practice to simulate and oppose ruminative memory biases. *Clinical Psychological Science* (**in press**).

[CR15] Hertel PT, Mor N, Ferrari C, Hunt O, Agrawal N (2014). Looking on the dark side: Rumination and cognitive bias modification. Clinical Psychological Science..

[CR16] Johnson SL, Joormann J, Gotlib IH (2007). Does processing of emotional stimuli predict symptomatic improvement and diagnostic recovery from major depression?. Emotion.

[CR17] Joormann J, Dkane M, Gotlib IH (2006). Adaptive and maladaptive components of rumination?. Behavior Therapy.

[CR18] Joormann J, Quinn ME (2014). Cognitive processes and emotion regulation in depression. Depression and Anxiety.

[CR19] Joormann J, Siemer M (2004). Memory accessibility, mood regulation, and dysphoria: Difficulties in repairing sad mood with happy memories?. Journal of Abnormal Psychology.

[CR20] Joormann J, Waugh CE, Gotlib IH (2015). Cognitive bias modification for interpretation in major depression: Effects on memory and stress reactivity. Clinical Psychological Science.

[CR21] Karpicke JD (2009). Metacognitive control and strategy selection: Deciding to practice retrieval during learning. Journal of Experimental Psychology: General.

[CR22] Karpicke JD, Grimaldi PJ (2012). Retrieval-based learning: A perspective for enhancing meaningful learning. Educational Psychology Review.

[CR23] Karpicke JD, Roediger HL (2008). The critical importance of retrieval for learning. Science.

[CR24] Mathews A, MacLeod C (2005). Cognitive vulnerability to emotional disorders. Annual Review Clinincal Psychology.

[CR25] Matt GE, Vazquez C, Campbell WK (1992). Mood-congruent recall of affectively toned stimuli: A meta-analytic review. Clinical Psychology Review.

[CR26] Menne-Lothmann C, Viechtbauer W, Höhn P, Kasanova Z, Haller SP, Drukker M, van Os J, Wichers M, Lau JYF (2014). How to boost positive interpretations? A meta-analysis of the effectiveness of Cognitive Bias Modification for interpretation. PLoS One.

[CR27] Nolen-Hoeksema S, Morrow JA (1991). Prospective study of depression and posttraumatic stress symptoms after a natural disaster: The 1989 Loma Prieta earthquake. Journal of Personality and Social Psychology.

[CR28] Nolen-Hoeksema S, Wisco BE, Lyubomirsky S (2008). Rethinking rumination. Perspectives on Psychological Science.

[CR29] Raes F, Hermans D, Eelen P (2003). De Nederlandstalige versie van de Ruminative Response Scale (RRS-NL) en de Rumination on Sadness Scale (RSS-NL). Gedragstherapie.

[CR30] Ridout N, Noreen A, Johal J (2009). Memory for emotional faces in naturally occurring dysphoria and induced sadness. Behaviour Research and Therapy.

[CR31] Rinck M, Becker ES (2005). A comparison of attentional biases and memory biases in women with social phobia and major depression. Journal of Abnormal Psychology.

[CR32] Roediger HL, Butler AC (2011). The critical role of retrieval practice in long-term retention. Trends in Cognitive Science.

[CR33] Rossi V, Pourtois G (2013). Negative affective state mimics effects of perceptual load on spatial perception. Emotion.

[CR34] Soderstrom NC, Bjork RA (2015). Learning versus performance: An integrative review. Perspectives on Psychological Science.

[CR35] Tran TB, Hertel PT, Joormann J (2011). Cognitive bias modification: Induced interpretive biases affect memory. Emotion.

[CR36] Treynor W, Gonzalez R, Nolen-Hoeksema S (2003). Rumination reconsidered: A psychometric analysis. Cognitive Therapy and Research.

[CR37] van der Does AJW (2002). BDI-II-NL. Handleiding. De Nederlandse versie van de Beck depression inventory.

[CR38] Vrijsen JN, Becker ES, Rinck M, van Oostrom I, Speckens A, Whitmer A, Gotlib IH (2014). Can memory bias be modified? The effects of an explicit cued-recall training in two independent samples. Cognitive Therapy and Research.

